# Supplementary material for: High-throughput determination of RNA tertiary contact thermodynamics by quantitative DMS chemical mapping
Source: Nucleic Acids Res. 2024 Jul 31;52(16):9953–65. doi: 10.1093/nar/gkae633 (PMC11381326; doi:10.1093/nar/gkae633)
Supplement: gkae633_Supplemental_Files [file gkae633_supplemental_files.zip › qDMS_ttr_paper_supplemental_w_response.docx]

Supplemental material for High-throughput determination of RNA tertiary contact thermodynamics by quantitative DMS chemical mapping

Bret Lange^1^, Ricardo G. Gil^1^, Gavin S. Anderson, and Joseph D. Yesselman^1^*

^1^Department of Chemistry, University of Nebraska, 639 North 12^th^ St, Lincoln, NE 68588, USA

*Corresponding author: jyesselm@unl.edu

Table of Contents

[Supplemental Figures 4](#_Toc169356824)

[Supplemental Figure 1: DMS-MaPseq mutation fractions as a function of sodium cacodylate buffer titration with 10 mM Mg^2+^. 5](#_Toc169356825)

[Supplemental Figure 2: Secondary structure of TL-knockout and TLR-knockout 6](#_Toc169356826)

[Supplemental Figure 3: 50 mM sodium cacodylate correctly captures the unformed state of the tetraloop/ tetraloop receptor contact. 7](#_Toc169356827)

[Supplemental Figure 4: Change of pH over time as a function of different sodium cacodylate buffer concentrations. 8](#_Toc169356828)

[Supplemental Figure 5: The three As in GAAA have similar reactivity profiles and can be averaged. 9](#_Toc169356829)

[Supplemental Figure 6: The three different destabilizing mutations 10](#_Toc169356830)

[Supplemental Figure 7: DMS-MaPseq mutation fractions for H1 insertion as a function of Mg^2+^ titration. 11](#_Toc169356831)

[Supplemental Figure 8: DMS-MaPseq mutation fractions for H2 insertion as a function of Mg^2+^ titration. 12](#_Toc169356832)

[Supplemental Figure 9: DMS-MaPseq mutation fractions for H3 insertion as a function of Mg^2+^ titration. 13](#_Toc169356833)

[Supplemental Figure 10: Direct correlation between GAAA mutation fraction at 40 mM and Mg^2+^. 14](#_Toc169356834)

[Supplemental Figure 11: The reactivity of each DMS active residue as a function of Mg^2+^ in the 3x3 motif. 15](#_Toc169356835)

[Supplemental Figure 12: The reactivity of each DMS active residue as a function of Mg^2+^ in the IRES motif. 16](#_Toc169356836)

[Supplemental Figure 13: The reactivity of each DMS active residue as a function of Mg^2+^ in the kink-turn motif. 17](#_Toc169356837)

[Supplemental Figure 14: GC flanking pair breaking open in UCUAAA_CAUGA and CCUACA_UACGG 18](#_Toc169356838)

[Supplemental Figure 16: Strong correlation between [Mg^2+^]_1/2_ and GAAA reactivity at 7.5 mM Mg^2+^. 21](#_Toc169356839)

[Supplemental Figure 17: Most Mg^2+^ concentrations yield high correlations with RNA-MaP ∆G measurements. 23](#_Toc169356840)

[Supplemental Figure 18: Hierarchical clustering of reactivity values for the tetraloop and the tetraloop receptor DMS active residues. 24](#_Toc169356841)

[Supplemental Figure 19: The sequence information for each variant in each color. All highlighted colors are mutations from the wild-type. Colors simply help show the mutations A is red, C is blue, G is yellow, and U is green. 25](#_Toc169356842)

[Supplemental Figure 21: reactivity average of C in UUCG, which is flipped out. 27](#_Toc169356843)

[Supplemental Figure 22: Helix randomization strategy to increase diversity. 28](#_Toc169356844)

# Supplemental Figures


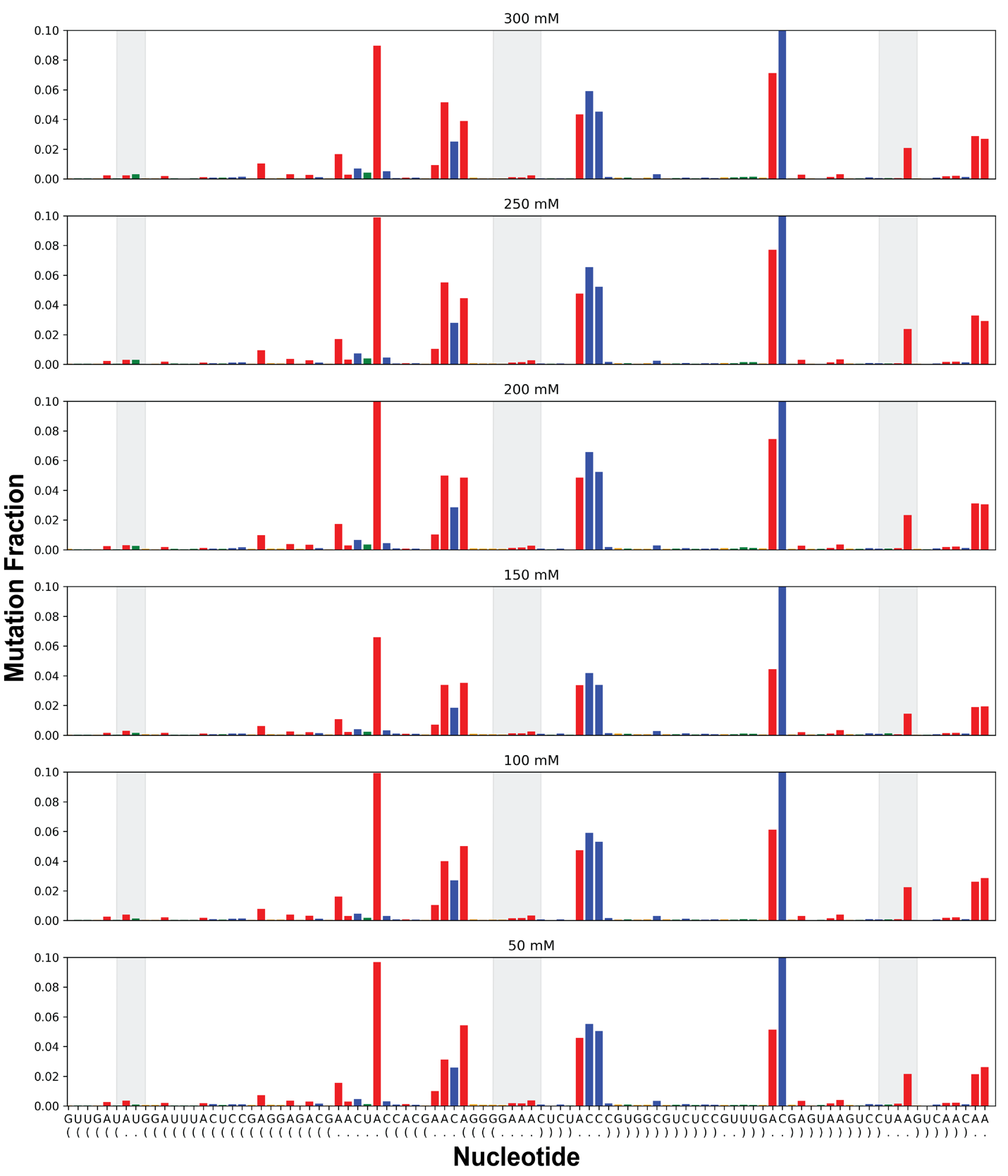


## Supplemental Figure 1: DMS-MaPseq mutation fractions as a function of sodium cacodylate buffer titration with 10 mM Mg^2+^.

Gray highlights the tetraloop in the center and the tetraloop receptor on the left and right.


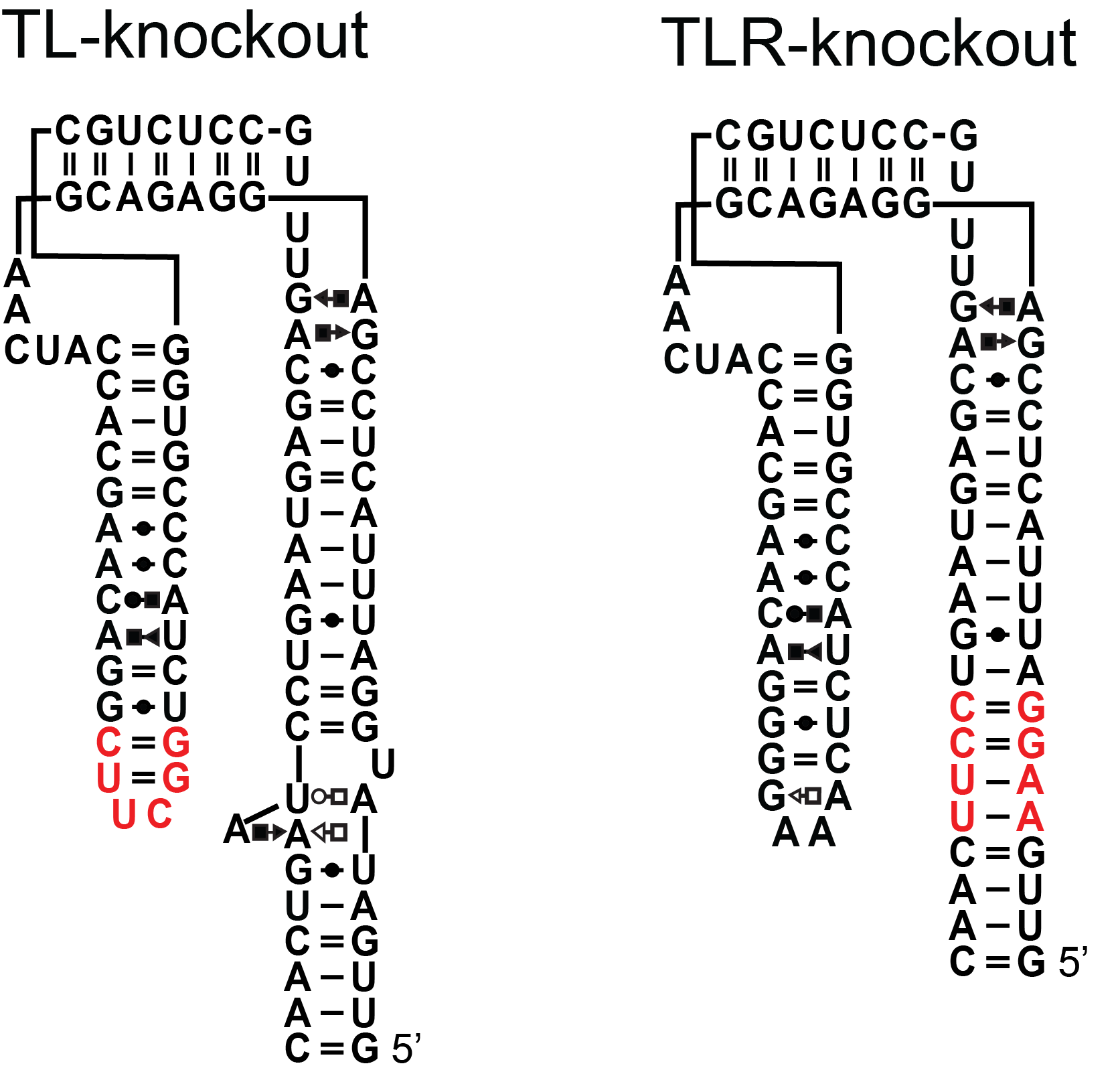


## Supplemental Figure 2: Secondary structure of TL-knockout and TLR-knockout

Red denotes mutations. In TL-knockout mutated the GAAA tetraloop to UUCG, in the TLR-knockout mutated the TLR to a Watson-Crick helix.


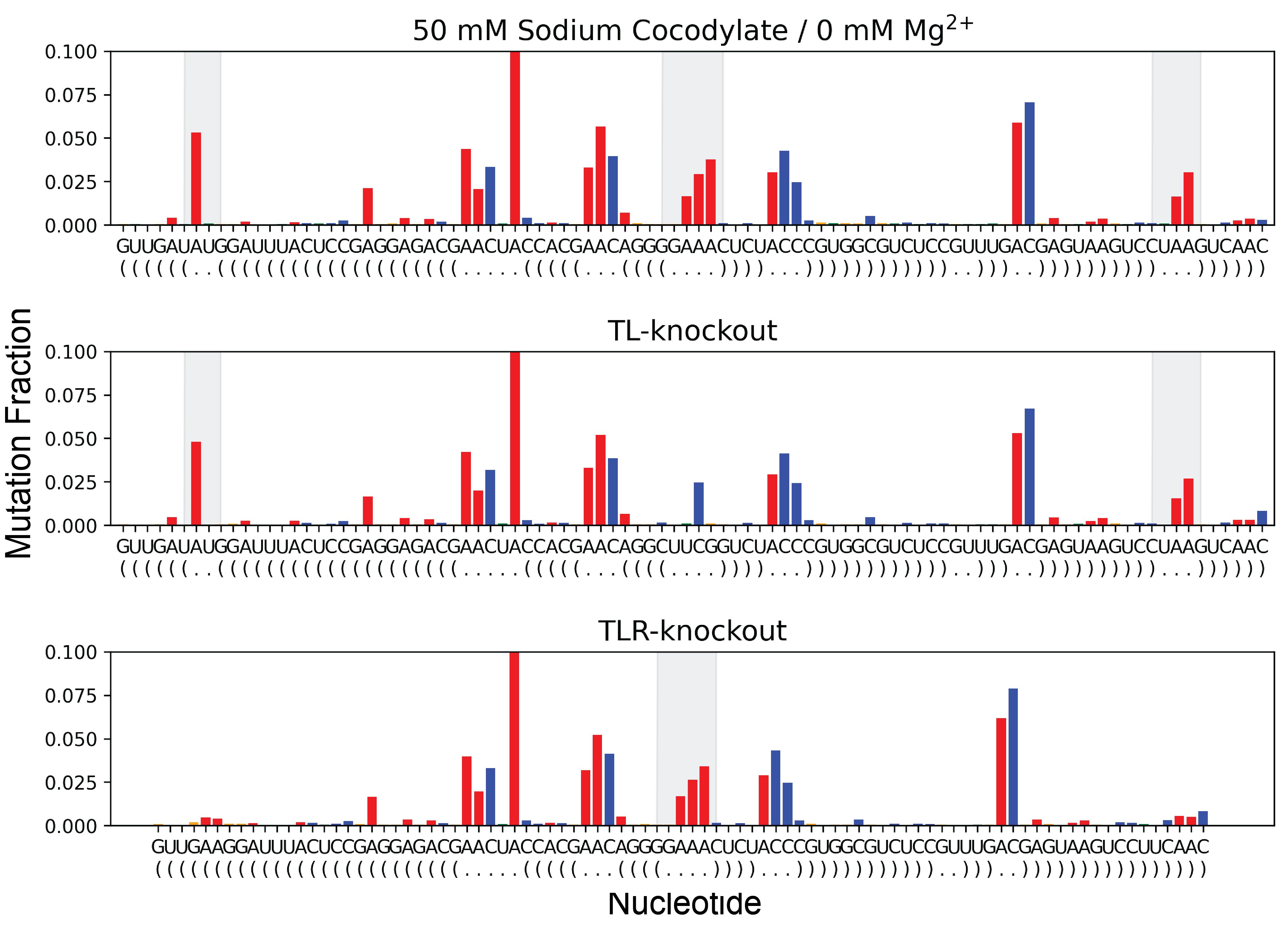


## Supplemental Figure 3: 50 mM sodium cacodylate correctly captures the unformed state of the tetraloop/ tetraloop receptor contact.

The wild-type without Mg^2+^ ions with 50 mM sodium cacodylate. The highlights are the tetraloop and tetraloop-receptor. Their reactivity closely matches the TL-knockout and TLR-knockout constructs.


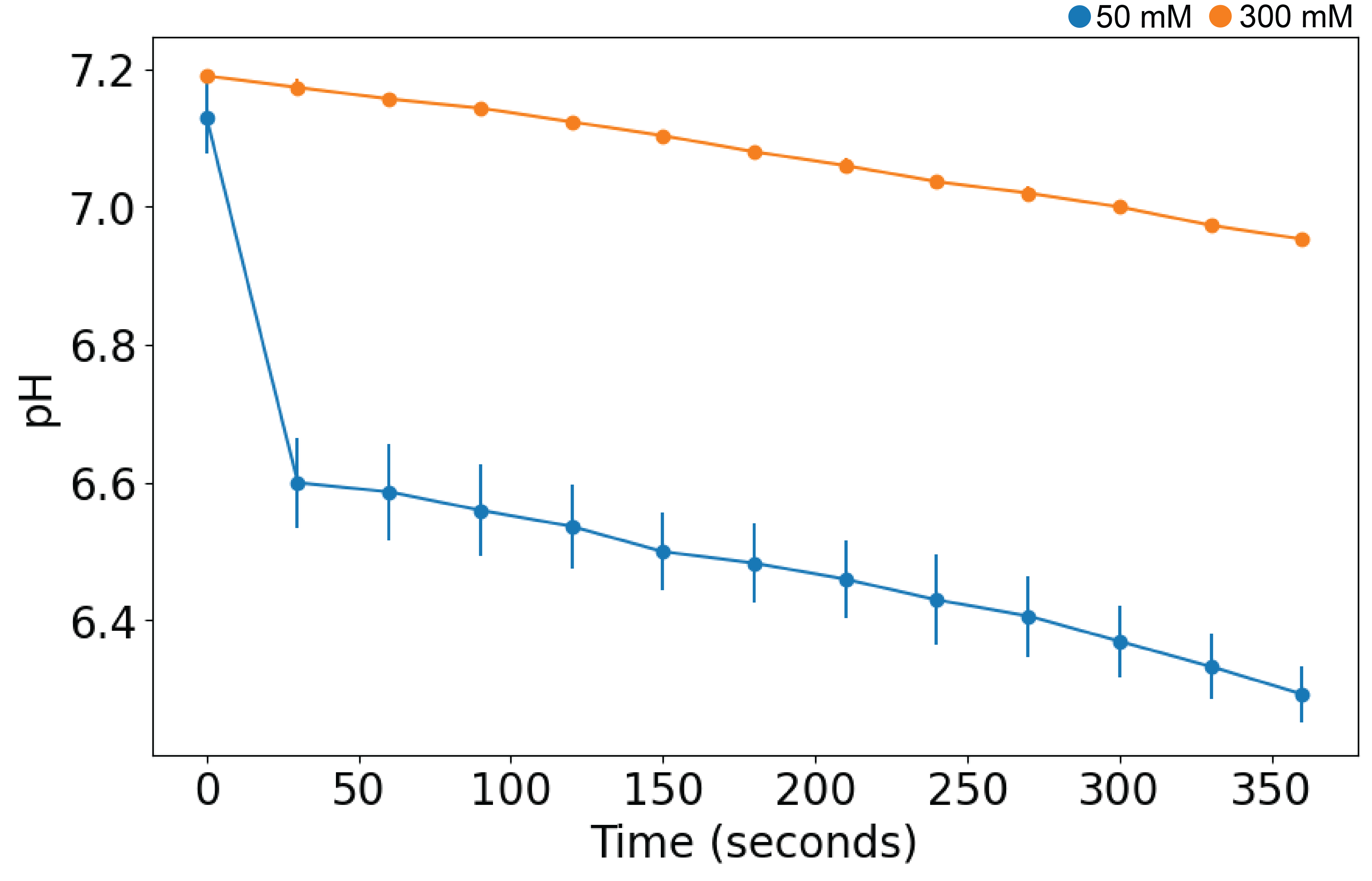


## Supplemental Figure 4: Change of pH over time as a function of different sodium cacodylate buffer concentrations.


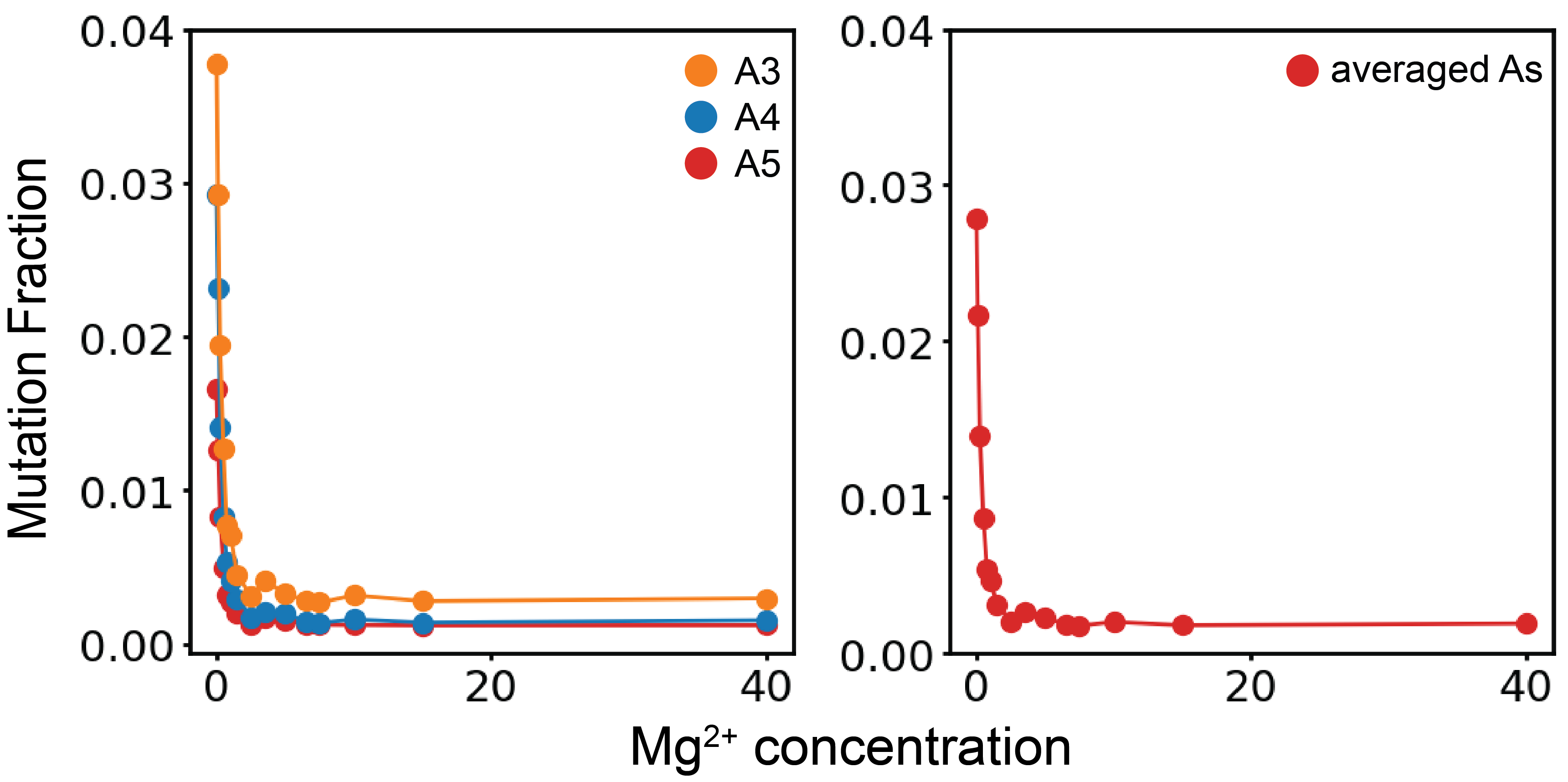


## Supplemental Figure 5: The three As in GAAA have similar reactivity profiles and can be averaged.

(Left) Shows the three adenines in the GAAA loop. Each has a near-identical reactivity profile as a function of Mg^2+^. (Right) Averaging the three adenines simplifies analysis and fitting.


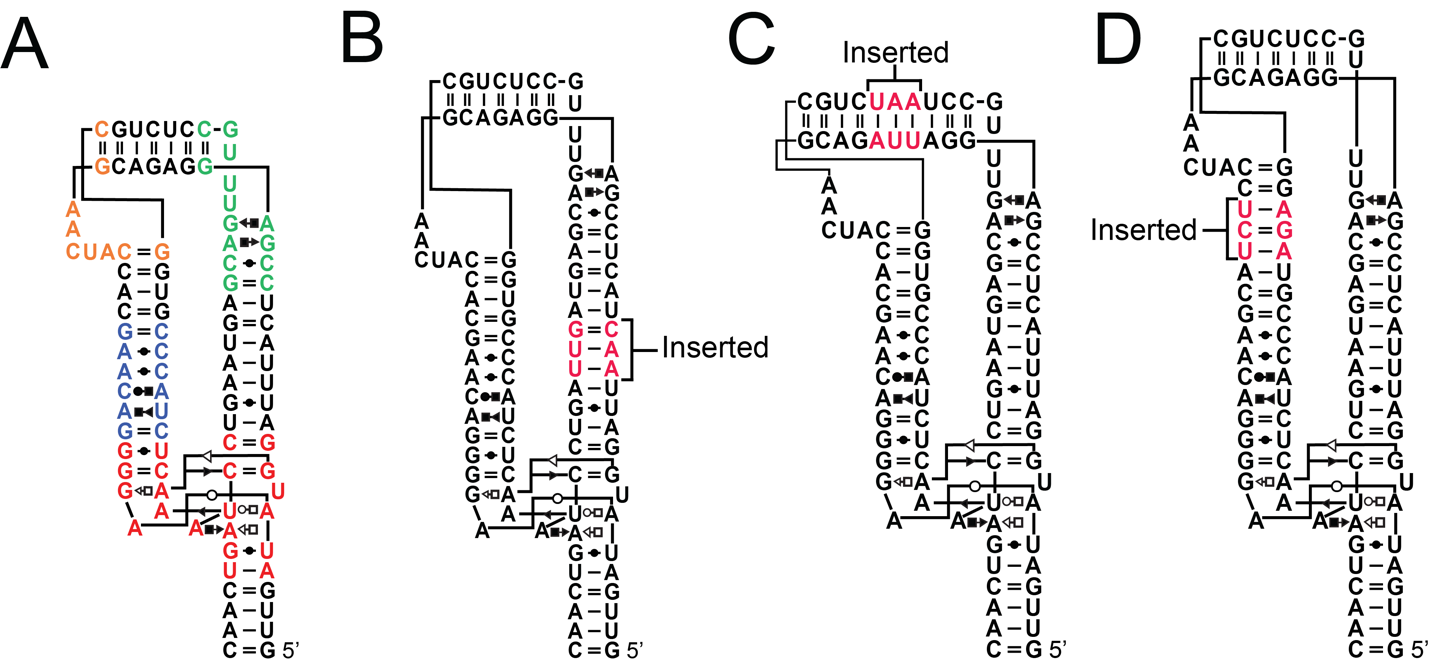


## Supplemental Figure 6: The three different destabilizing mutations

Shows the three different 3 bp insertions to destabilize the TL/TLR tertiary contact. (A) Wild-type (B) Insertion into helix 1 (H1). (C) Insertion into helix 2 (H2). (D) Insertion into helix 3 (H3).


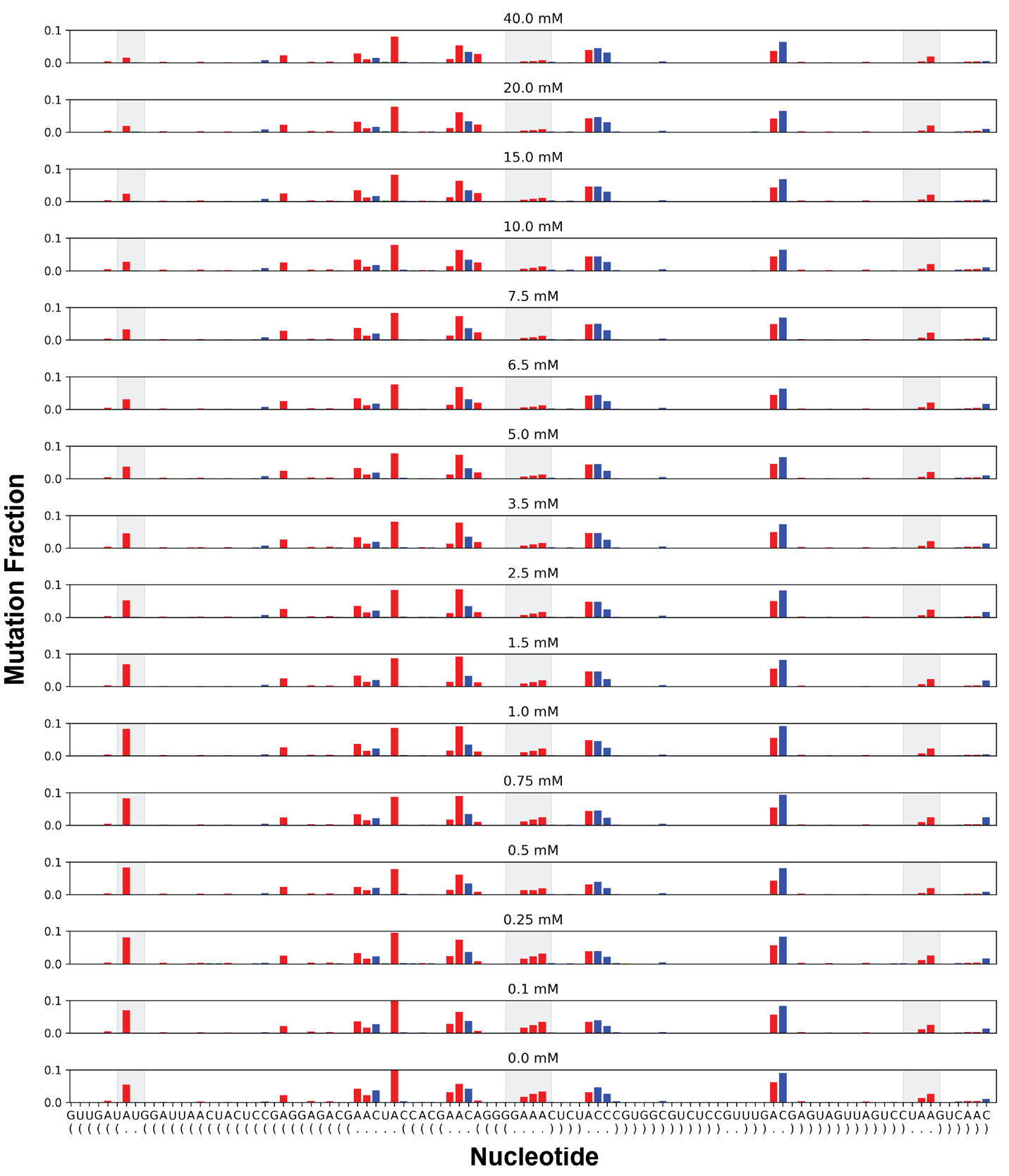


## Supplemental Figure 7: DMS-MaPseq mutation fractions for H1 insertion as a function of Mg^2+^ titration.


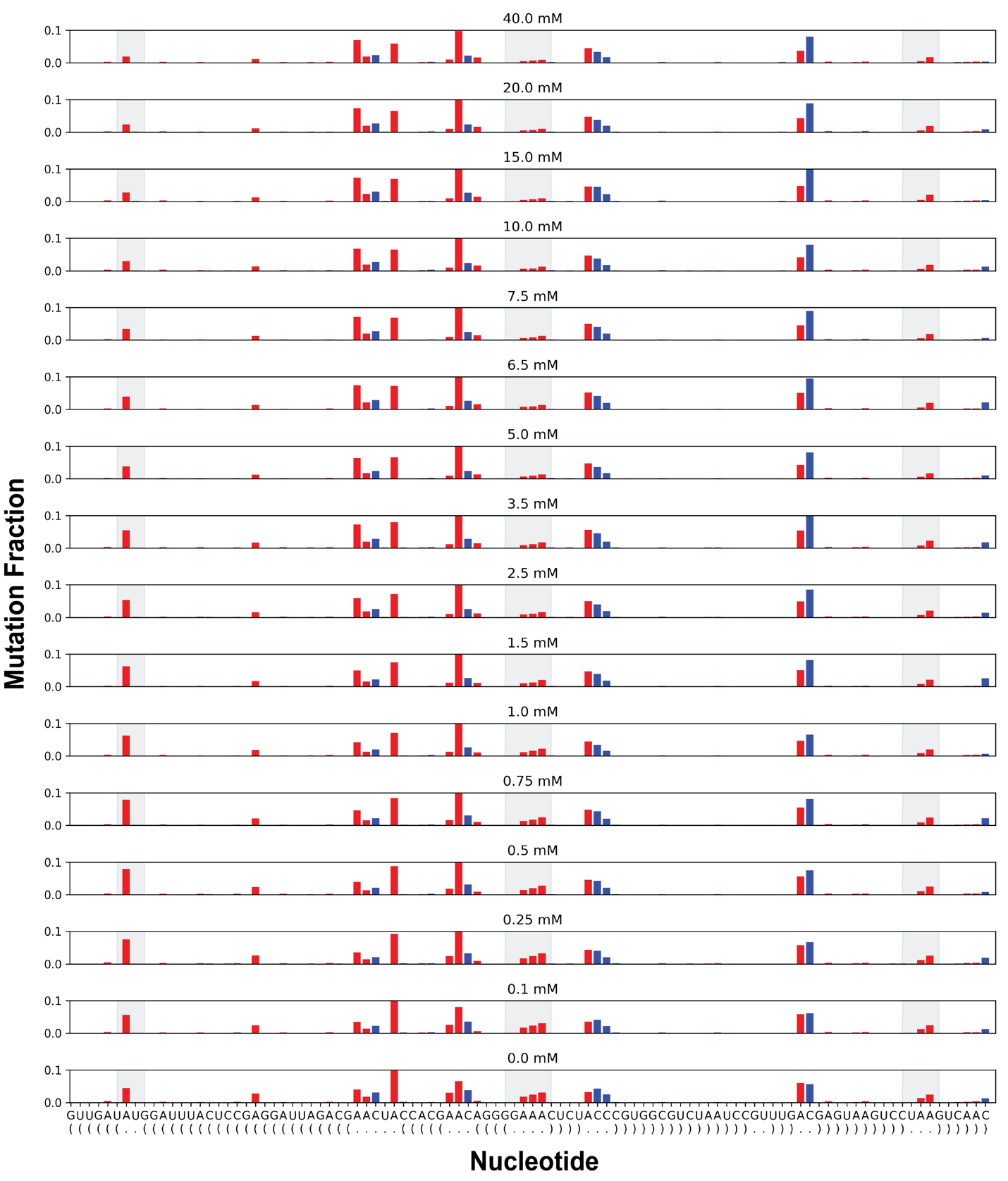


## Supplemental Figure 8: DMS-MaPseq mutation fractions for H2 insertion as a function of Mg^2+^ titration.

#####
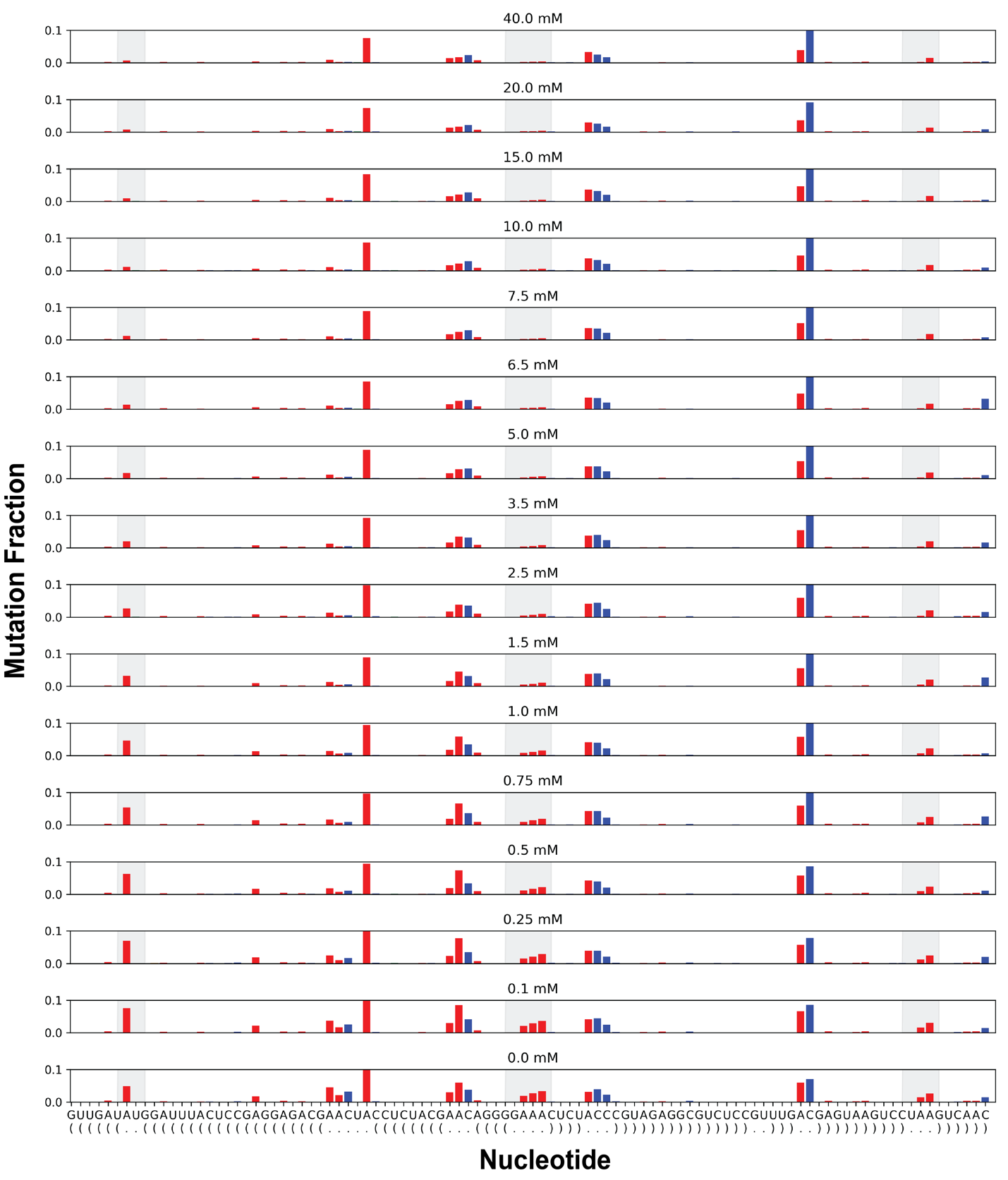


## Supplemental Figure 9: DMS-MaPseq mutation fractions for H3 insertion as a function of Mg^2+^ titration.


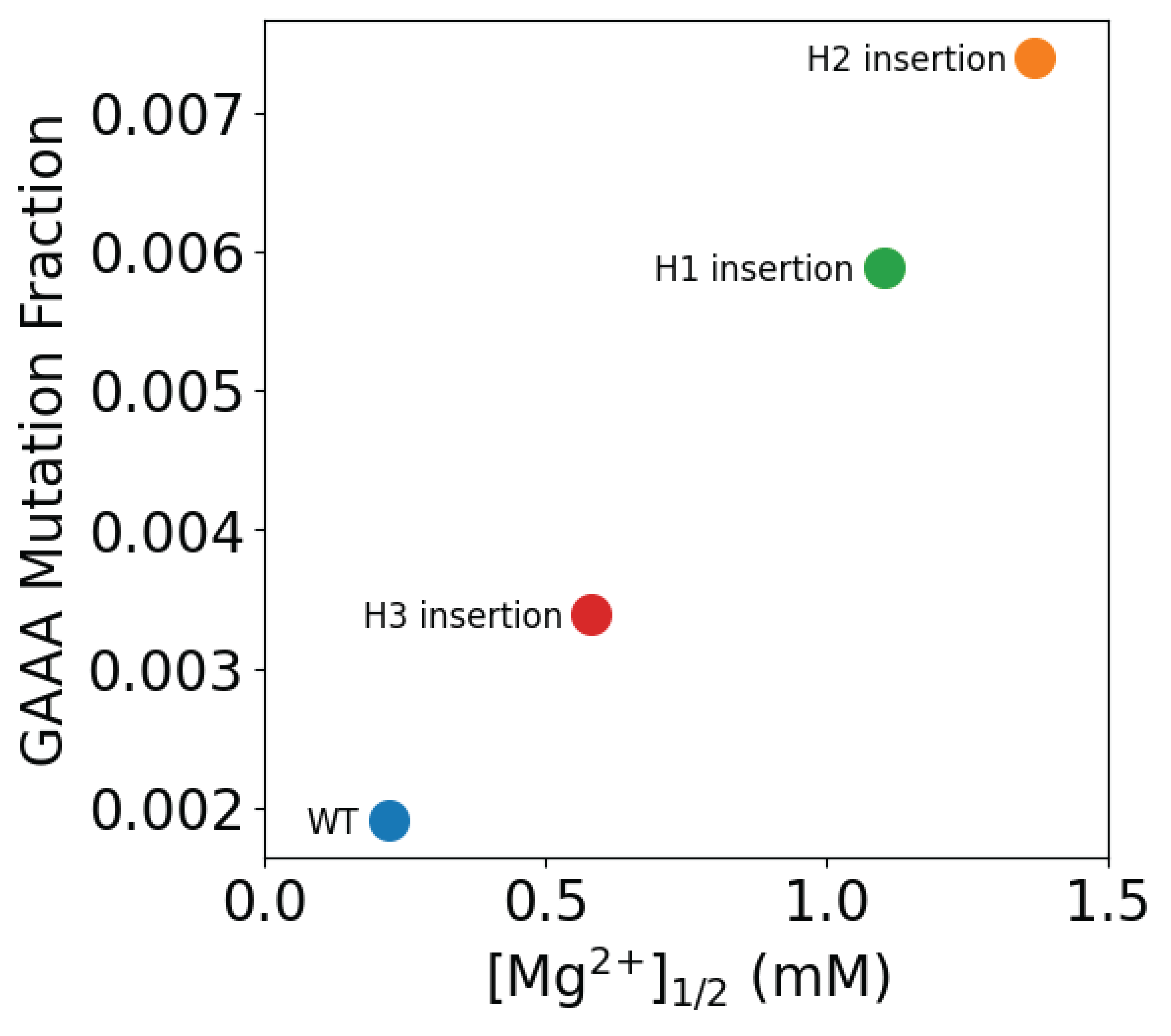


## Supplemental Figure 10: Direct correlation between GAAA mutation fraction at 40 mM and Mg^2+^.


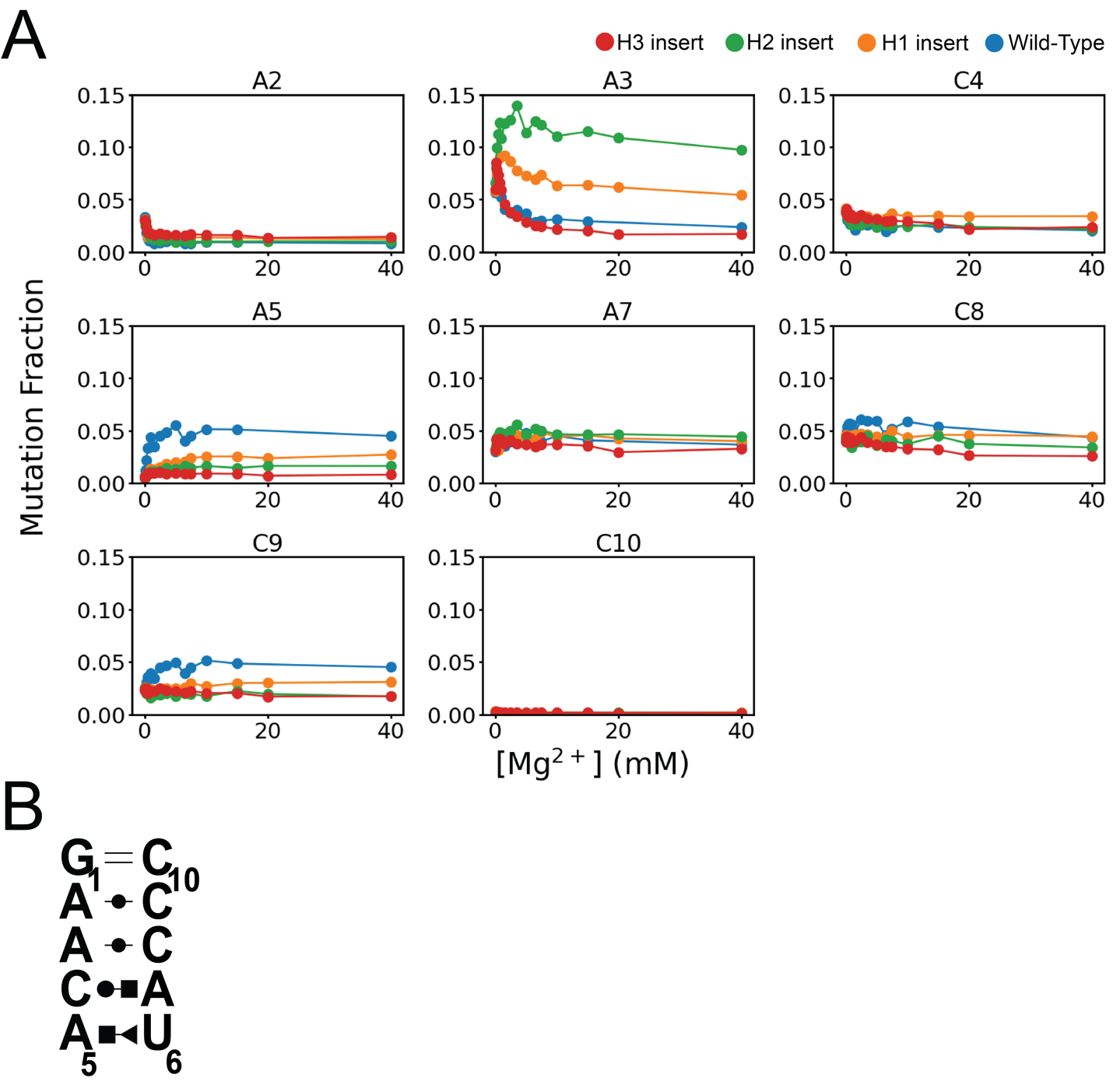


## Supplemental Figure 11: The reactivity of each DMS active residue as a function of Mg^2+^ in the 3x3 motif.


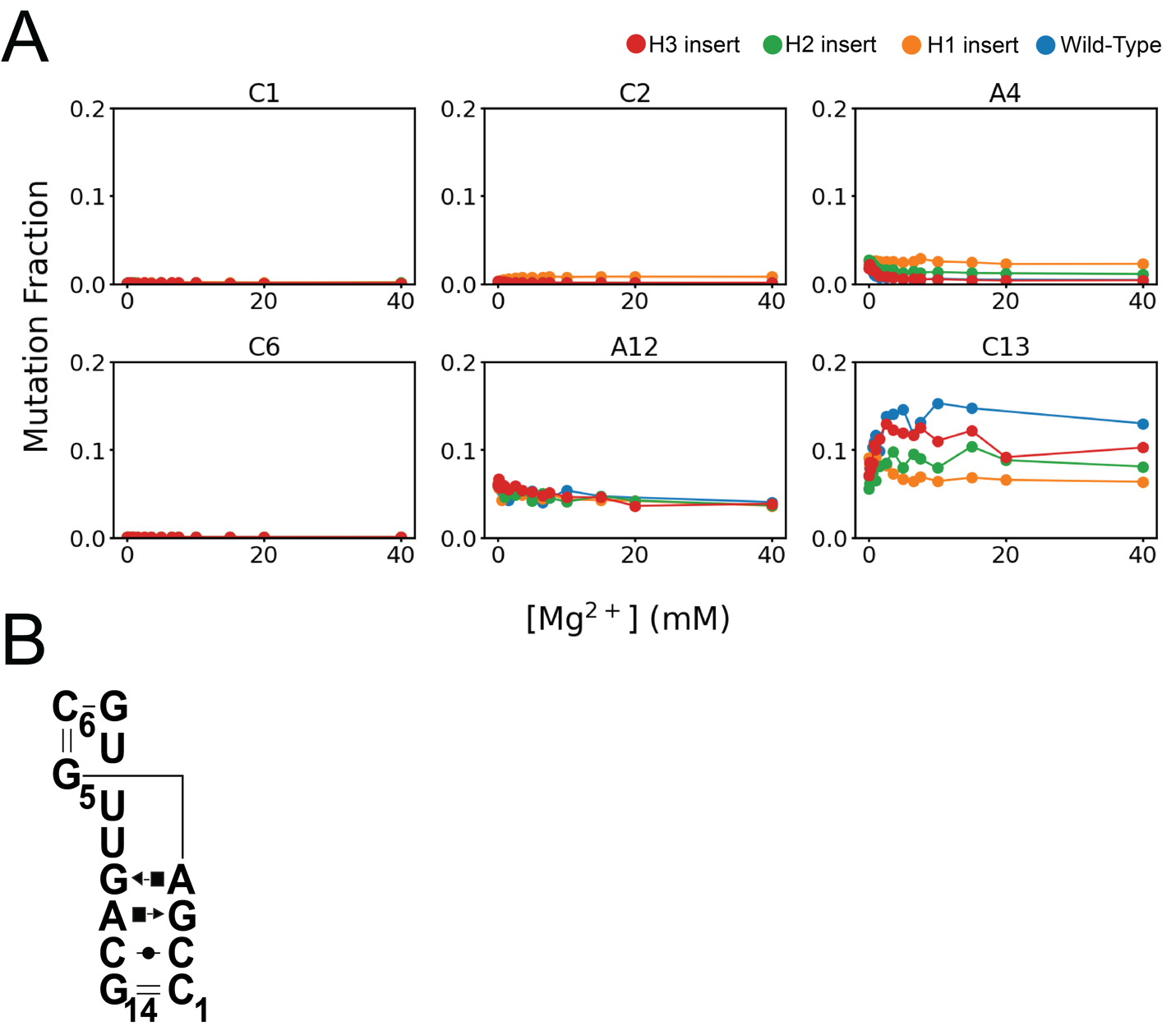


## Supplemental Figure 12: The reactivity of each DMS active residue as a function of Mg^2+^ in the IRES motif.


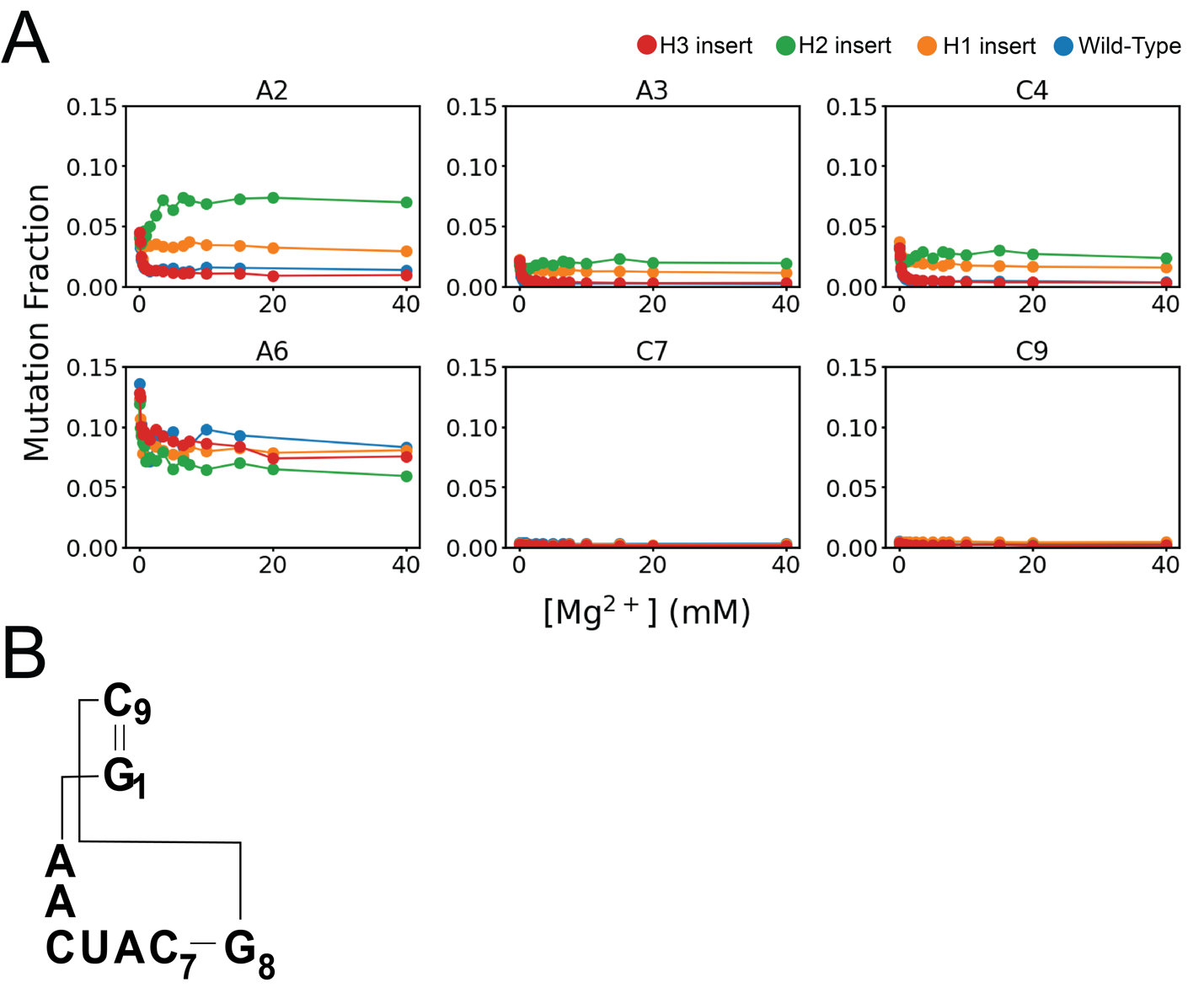


## Supplemental Figure 13: The reactivity of each DMS active residue as a function of Mg^2+^ in the kink-turn motif.


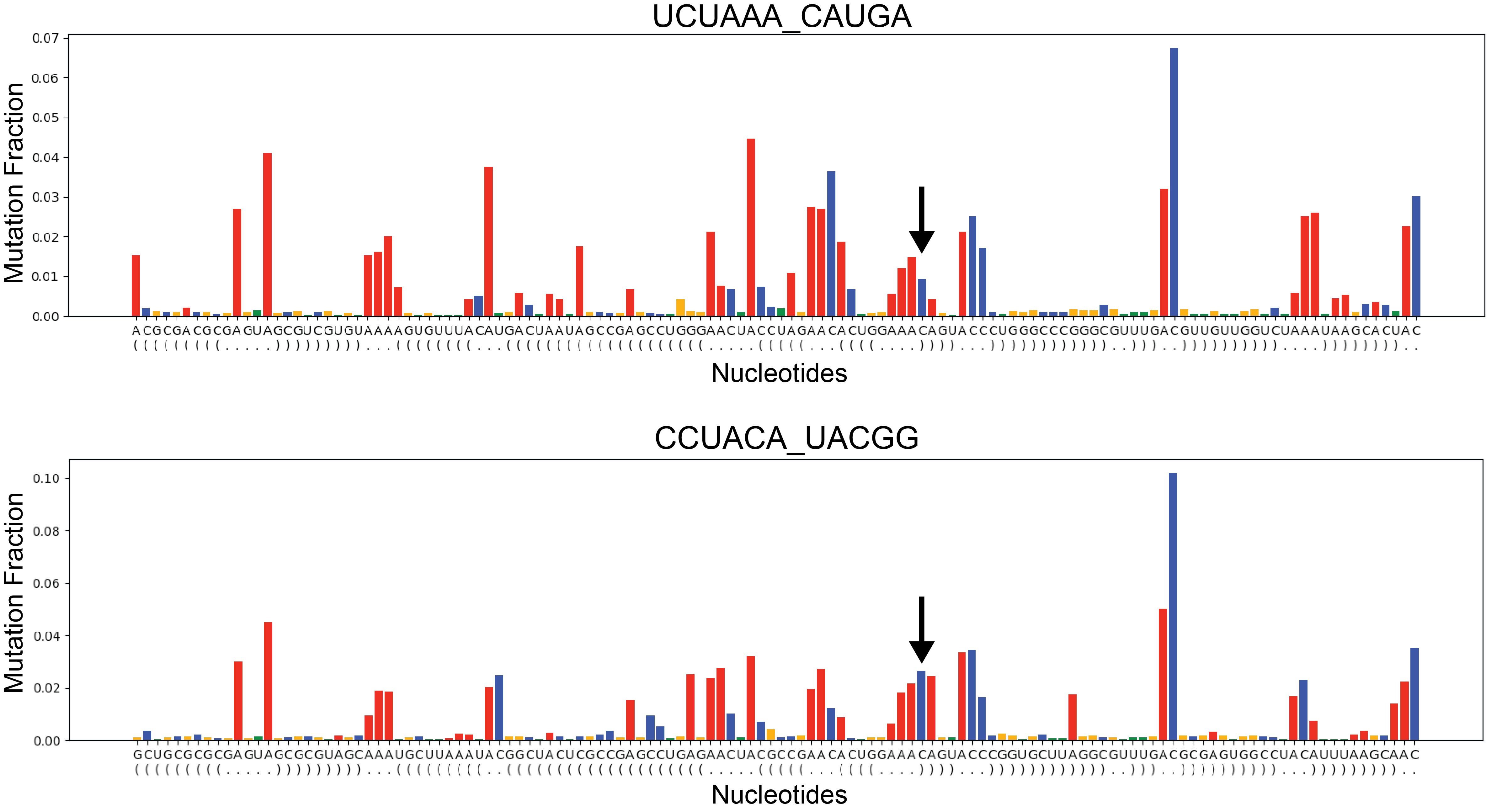


## Supplemental Figure 14: GC flanking pair breaking open in UCUAAA_CAUGA and CCUACA_UACGG


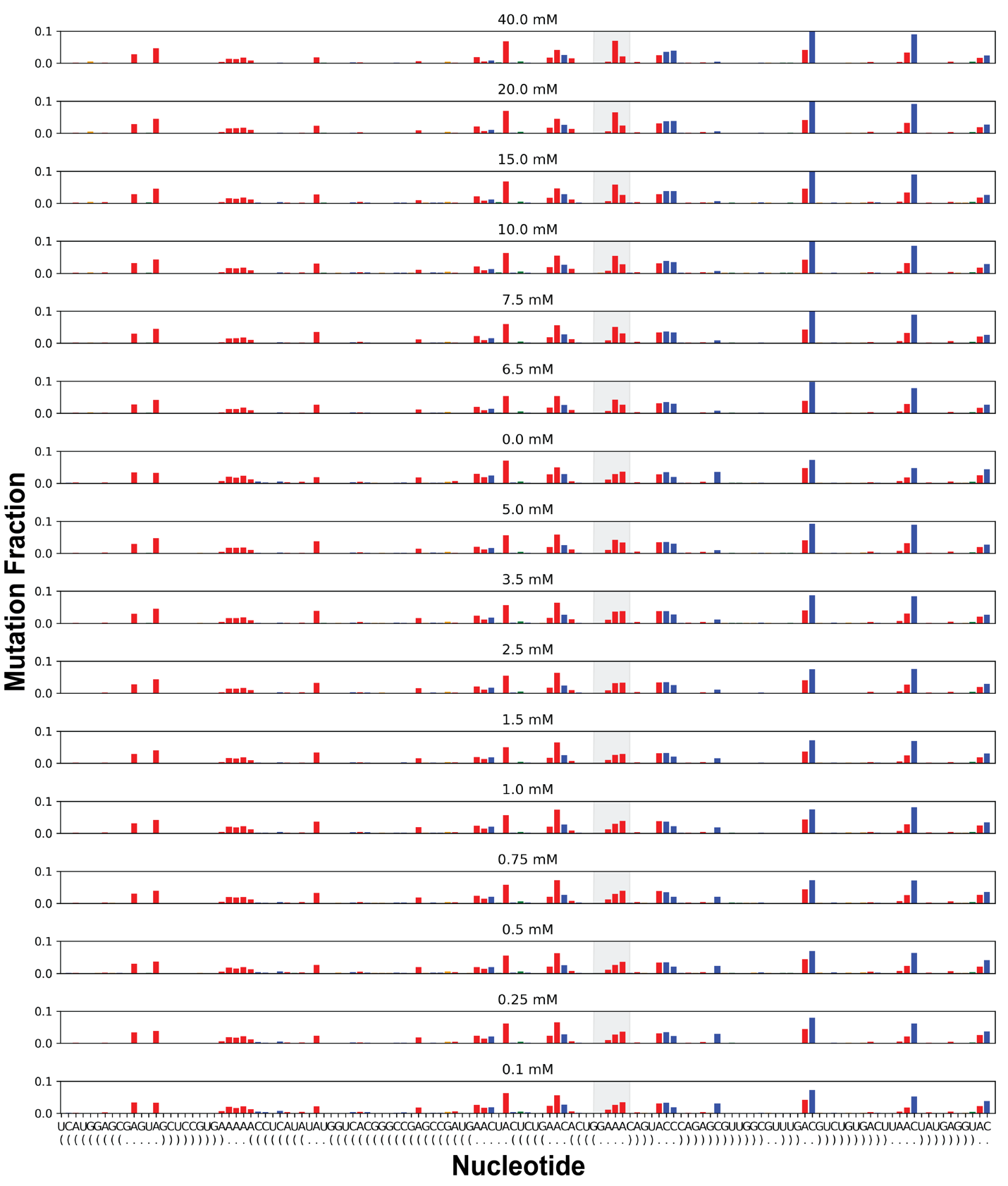


Supplemental Figure 15: CUUAAC_UAUGG titration does not have the GAAA reactivity decrease.

Instead, only A3 and A5 go down while A2 increases in reactivity which is not consistent with our model of TL/TLR binding.


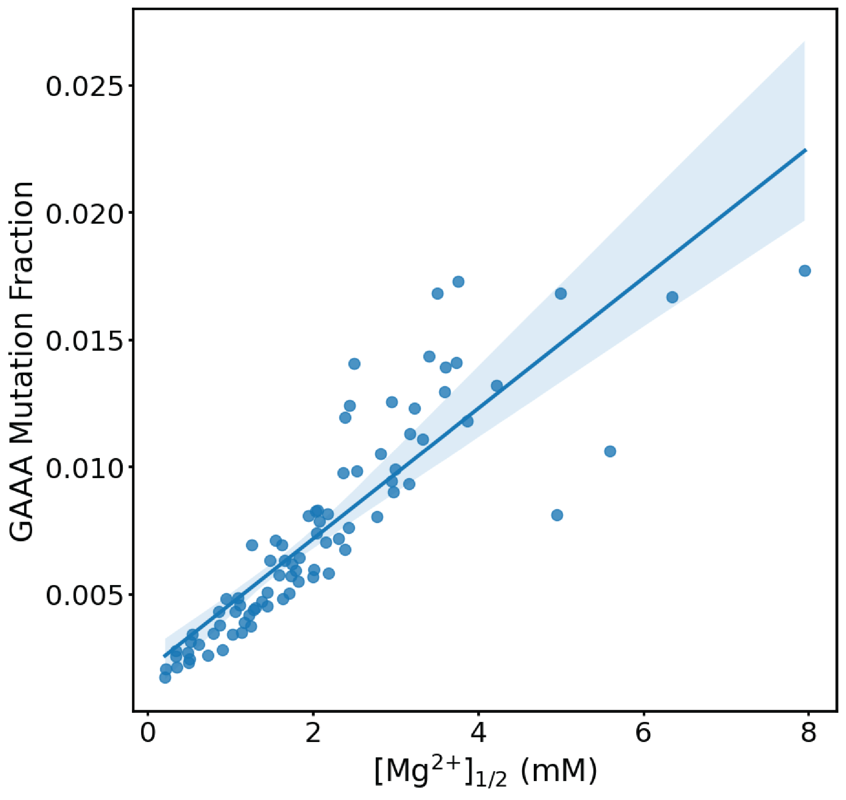


## Supplemental Figure 16: Strong correlation between [Mg^2+^]_1/2_ and GAAA reactivity at 7.5 mM Mg^2+^.


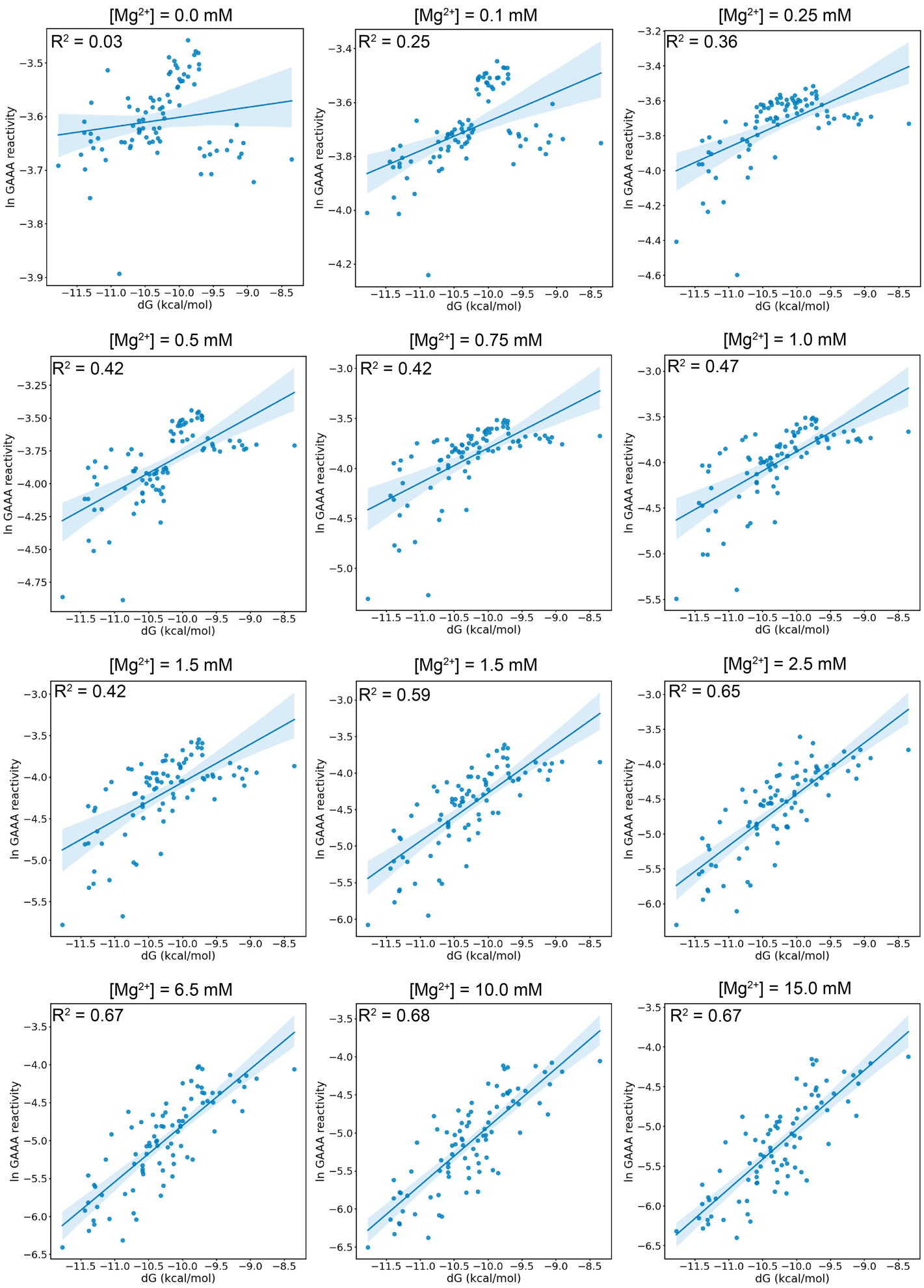


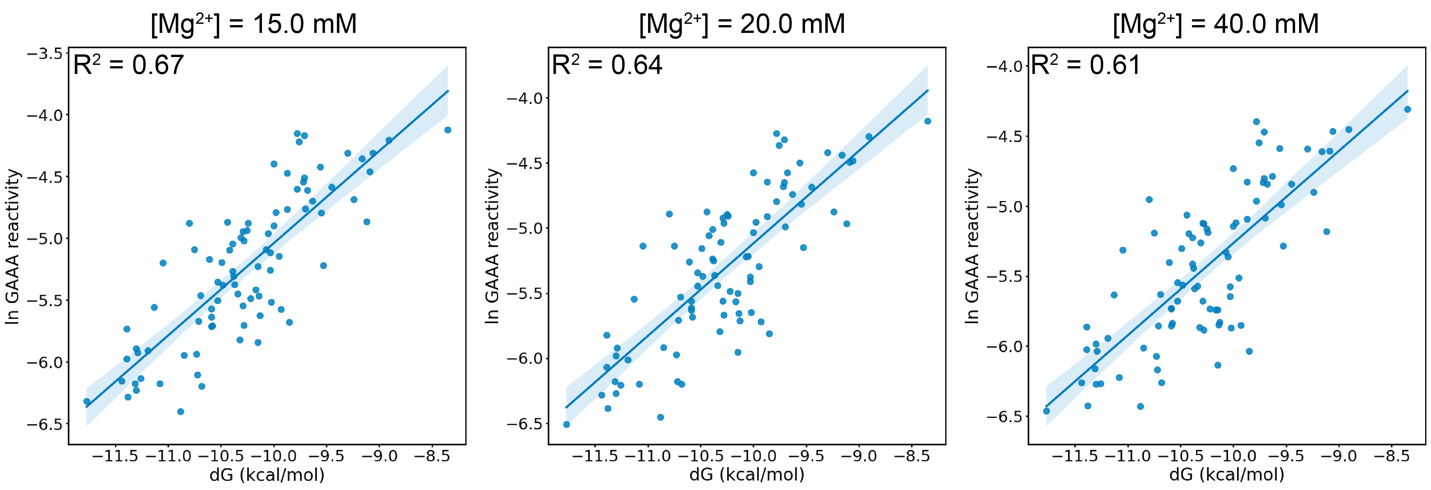


## Supplemental Figure 17: Most Mg^2+^ concentrations yield high correlations with RNA-MaP ∆G measurements.


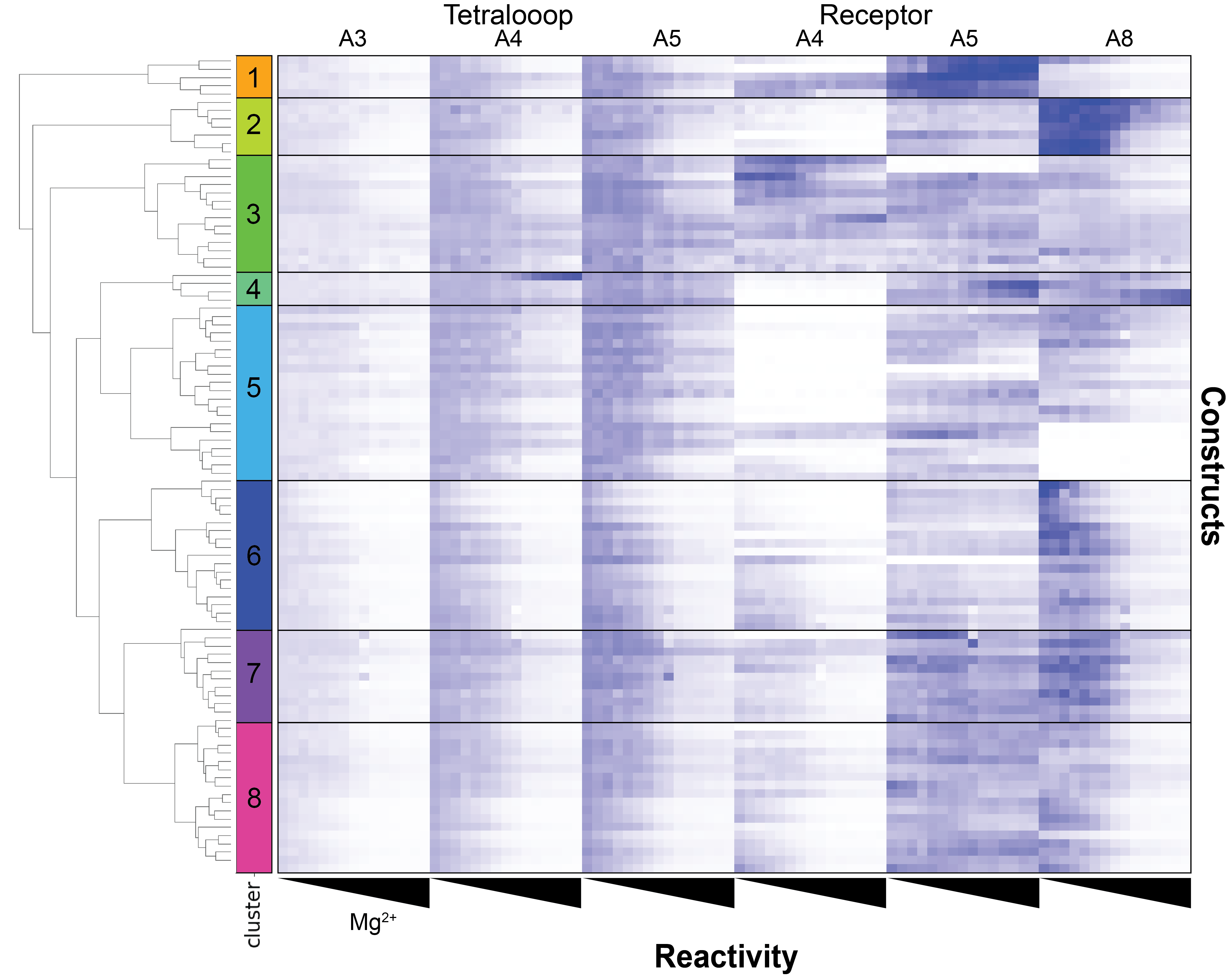


## Supplemental Figure 18: Hierarchical clustering of reactivity values for the tetraloop and the tetraloop receptor DMS active residues.


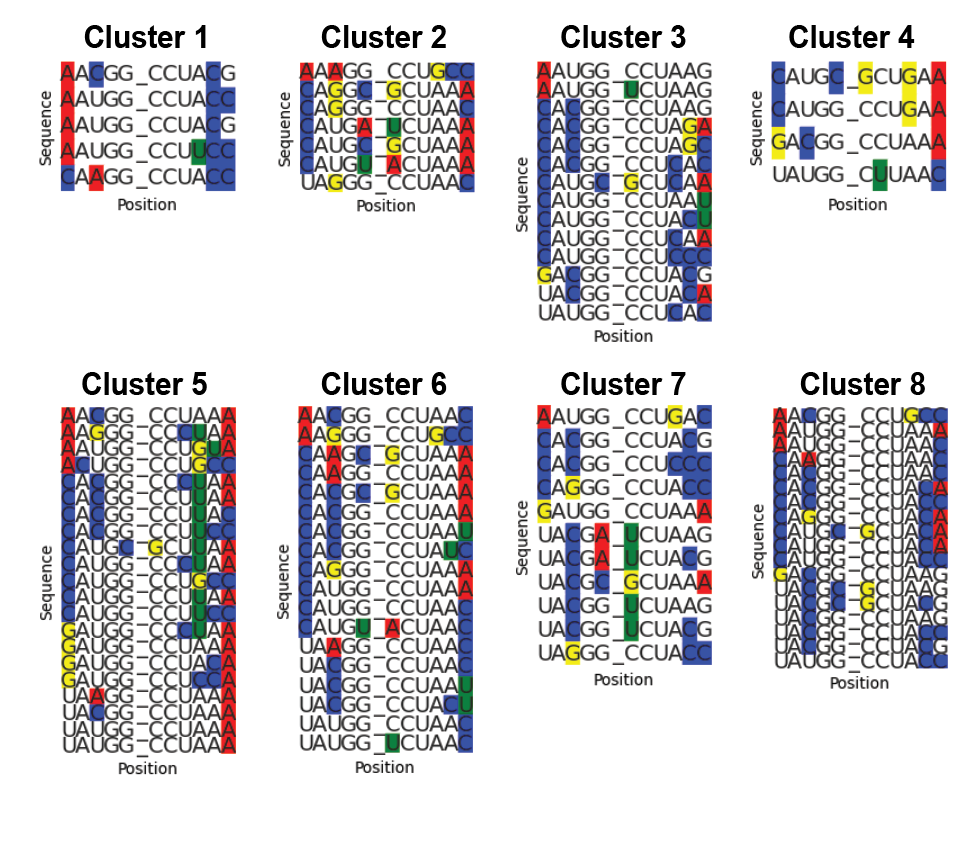


## Supplemental Figure 19: The sequence information for each variant in each color. All highlighted colors are mutations from the wild-type. Colors simply help show the mutations A is red, C is blue, G is yellow, and U is green.


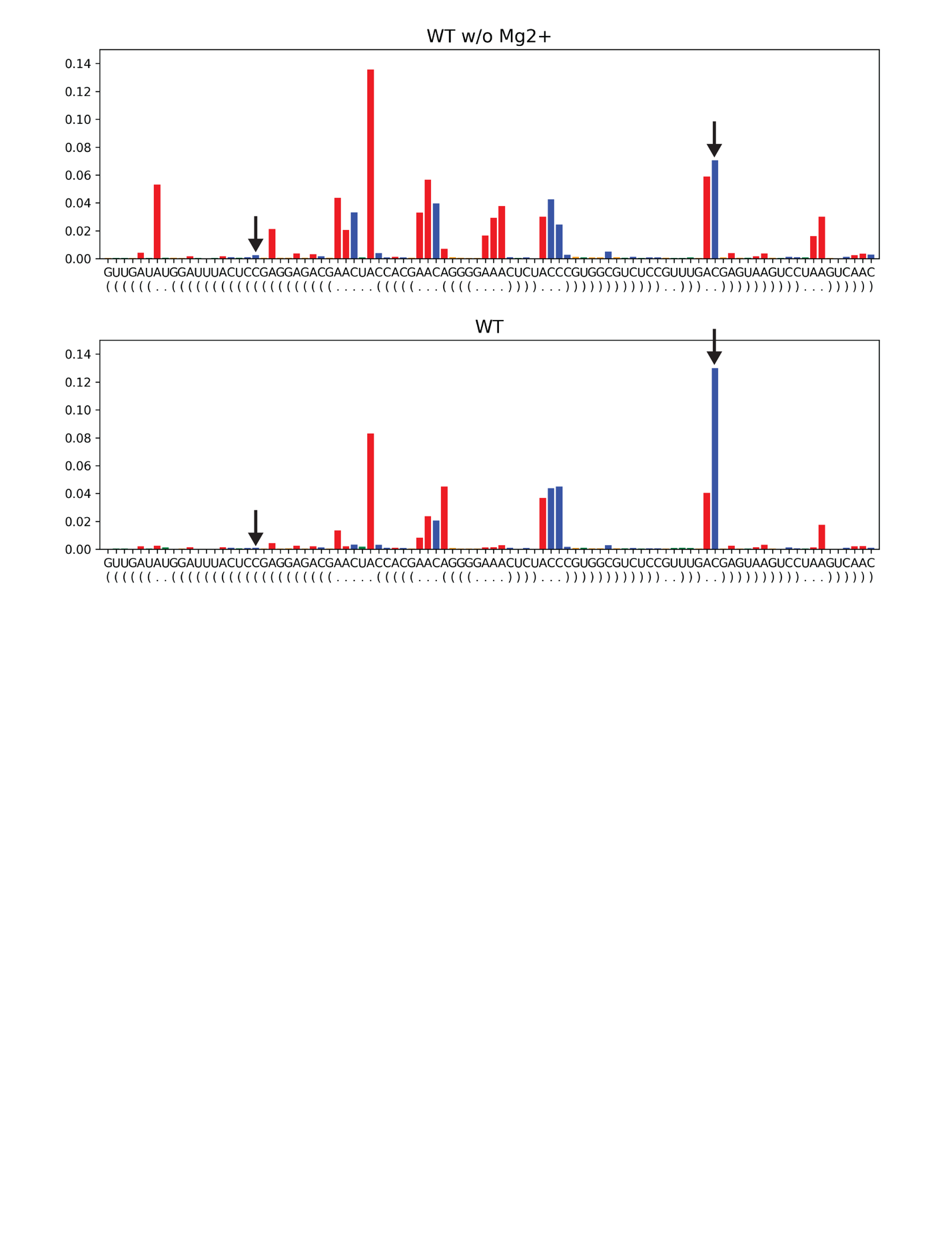
Supplemental Figure 20: Reactivity profile of kink turn of C-C pair like the C-C mismatch in the CCUAAC_CAUGG TLR variant.


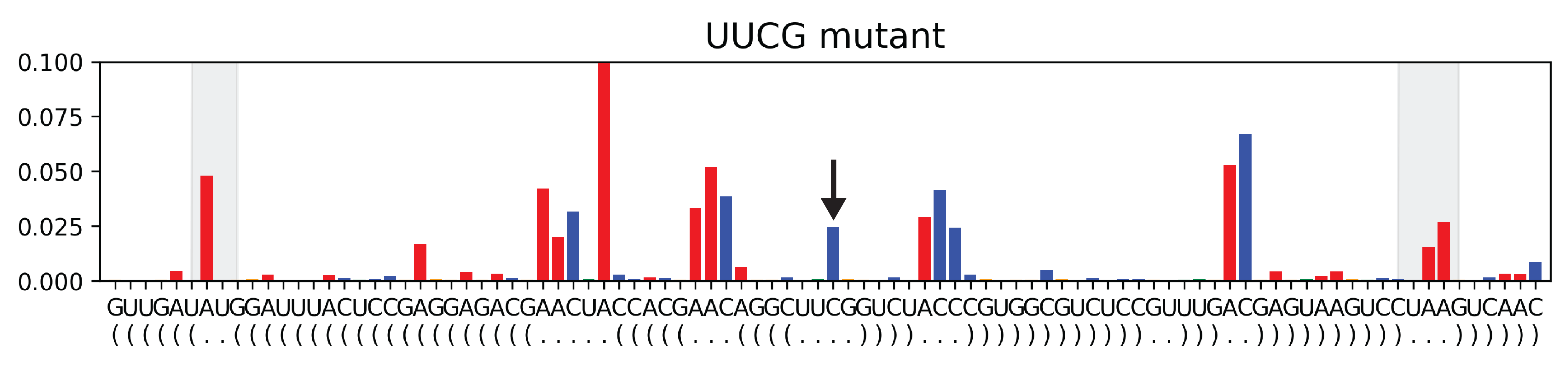


## Supplemental Figure 21: reactivity average of C in UUCG, which is flipped out.


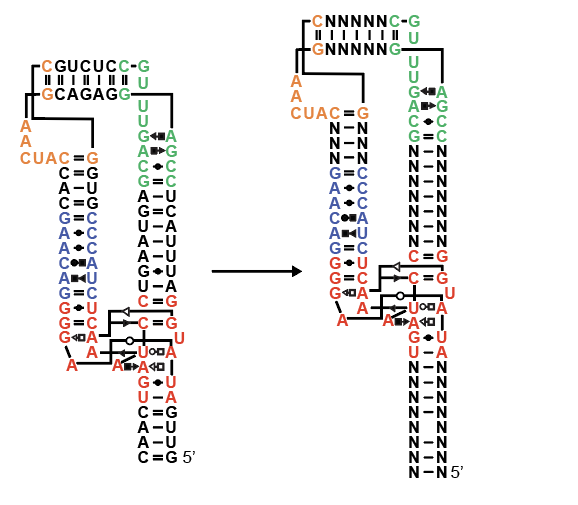


## Supplemental Figure 22: Helix randomization strategy to increase diversity.
